# Draft Genome Sequence of *Ralstonia solanacearum* Strain Rs-T02, Which Represents the Most Prevalent Phylotype in Guangxi, China

**DOI:** 10.1128/genomeA.00241-16

**Published:** 2016-04-14

**Authors:** Chengwu Zou, Kaihao Wang, Jiaorong Meng, Gaoqing Yuan, Wei Lin, Haowen Peng, Qiqin Li

**Affiliations:** College of Agriculture, Guangxi University, Nanning, China

## Abstract

*Ralstonia solanacearum* strain Rs-T02 was originally isolated from a bacterial wilt of tomato plant in Nanning City of Guangxi Province, China. It represents the most prevalent phylotype in Guangxi. Here, we present the draft genome sequence of this strain, which comprises 5,225 genes and 5,976,011 nucleotides with an average G+C content of 66.79%. There are 968 different genes between this isolate and the previously reported genome sequence of *Ralstonia solanacearum* GMl l000 (race l, biovar 3, phylotype I), and the genome sequence information of this isolate may be useful for comparative genomic studies to determine the genetic diversity in this species.

## GENOME ANNOUNCEMENT

*Ralstonia solanacearum* (Smith) Yabuuchi et al. (formerly called *Pseudomonas solanacearum*) is a soilborne bacterial pathogen that is a major limiting factor in the production of many crop plants around the world. This organism is the causal agent of brown rot of potato; bacterial wilt or southern wilt of tomato, tobacco, eggplant, and some ornamentals; and Moko disease of banana (1). *Ralstonia solanacearum* is a widely distributed pathogen found in tropical, subtropical, and some temperate regions of the world (2). The species as a whole has a very broad host range and infects hundreds of species in many plant families. The majority of hosts are dicots, with the major exception being bananas and plantains. Most economically important host plants are found in the *Solanaceae* or nightshade family (3). The specific host ranges and distributions of *Ralstonia solanacearum* depend on the race and to some degree the biovar of the pathogen. These host ranges and distributions have been changing in recent years. To facilitate a greater understanding of genetic diversity in this species, we sequenced the genome of *Ralstonia solanacearum* strain Rs-T02, which represents the most prevalent phylotype in Guangxi, China.

This strain was isolated from Nanning City of Guangxi Province, China. Gram staining and PCR amplification and sequencing of 16s rRNA genes were used to confirm the isolate as *Ralstonia solanacearum*, temporarily named Rs-T02. Genomic DNA was isolated from culture using a standard genomic DNA purification protocol (4). Two DNA libraries were constructed: a paired-end library with an insert size of 500 bp and a mate-pair library with an insert size of 5 kb. The 500-bp library and the 5-kb library were sequenced using an Illumina HiSeq2500 with a PE125 strategy to produce 2,228 Mb of clean data, representing >200-fold coverage of the genome. Genome assembly was performed using the SOAPdenovo package under default parameters (5, 6). The resulting draft genome for *Ralstonia solanacearum* strain Rs-T02 had 15 scaffolds covering 5,976,011 bp, with 66.79% GC content. Genome annotation was performed using the GeneMarkS (http://topaz.gatech.edu/GeneMark/), with which 5,225 putative protein-coding genes were predicted. tRNAs (60 tRNA sequences) were identified using tRNAscan-SE (7); rRNA genes (4 5S rRNAs, 5 16S rRNAs, and 4 23S rRNAs) were predicted with rRNAmmer (8); and small RNAs (sRNAs) (0 sRNA sequence) were predicted by the BLAST against the Rfam (9) database. Comparison of this genome (using MUMmer and LASTZ) to that of *Ralstonia solanacearum* GMll000 (race l, biovar3, phylotype I) (GenBank accession numbers NC_003295.1 and NC_003296.1) revealed 100 deletions, 75 insertions, and 228 inversions, indicating that there is substantial genetic diversity. Further analysis of this genome in comparison to other sequenced *Ralstonia solanacearum* genomes may provide insight into the genetic diversity in this species.

### Nucleotide sequence accession numbers.

This whole-genome shotgun project has been deposited at DDBJ/EMBL/GenBank under the accession number LKVH00000000. The version described in this paper is version LKVH01000000.
